# Transcriptional Activity of the Bacterial Replication Initiator DnaA

**DOI:** 10.3389/fmicb.2021.662317

**Published:** 2021-06-01

**Authors:** Inoka P. Menikpurage, Kristin Woo, Paola E. Mera

**Affiliations:** Department of Microbiology, University of Illinois at Urbana-Champaign, Urbana, IL, United States

**Keywords:** DnaA, replication initiation, transcription factor, cell cycle, sporulation, DNA repair, autoregulation

## Abstract

In bacteria, DnaA is the most conserved DNA replication initiator protein. DnaA is a DNA binding protein that is part of the AAA+ ATPase family. In addition to initiating chromosome replication, DnaA can also function as a transcription factor either as an activator or repressor. The first gene identified to be regulated by DnaA at the transcriptional levels was *dnaA*. DnaA has been shown to regulate genes involved in a variety of cellular events including those that trigger sporulation, DNA repair, and cell cycle regulation. DnaA’s dual functions (replication initiator and transcription factor) is a potential mechanism for DnaA to temporally coordinate diverse cellular events with the onset of chromosome replication. This strategy of using chromosome replication initiator proteins as regulators of gene expression has also been observed in archaea and eukaryotes. In this mini review, we focus on our current understanding of DnaA’s transcriptional activity in various bacterial species.

## Introduction

**FIGURE 1:**
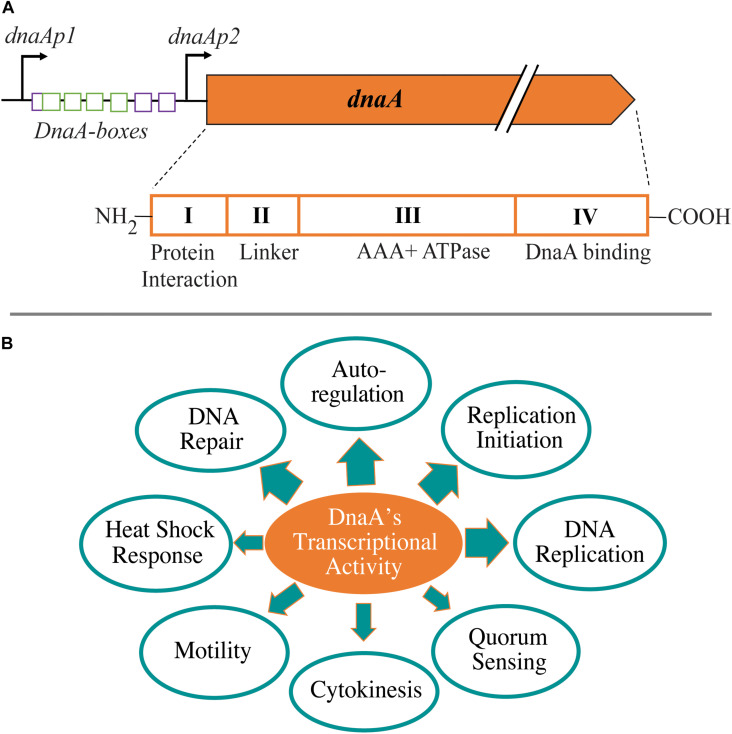
Cellular impact from transcriptional activity of DnaA. **(A)** Schematic of the promoter region of *dnaA* in *Escherichia coli* and conserved domains of the DnaA protein. Within the promoter region, purple boxes depict ATP-specific DnaA boxes and green boxes have no nucleotide-bound DnaA specificity. **(B)** Cellular processes from different bacterial species shown to be influenced by DnaA’s transcriptional activity. Thickness of arrow is an approximate representation of the amount of evidence that exists for that particular process.

**FIGURE 2:**
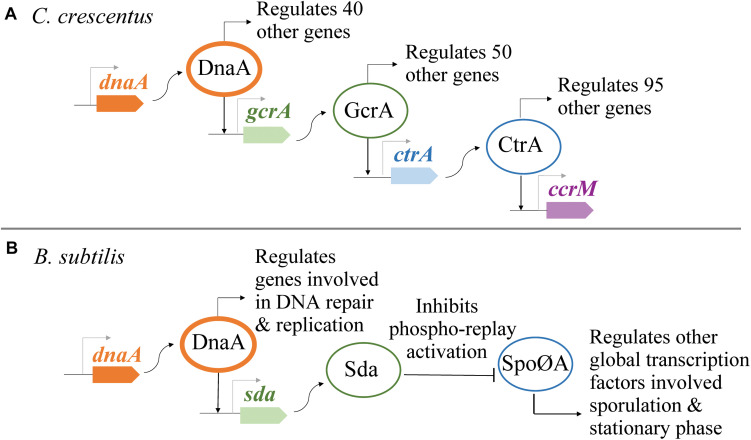
DnaA regulates the transcription of other global regulators. **(A)** In *Caulobacter crescentus*, DnaA is part of a genetic network responsible for regulating the forward progression of the cell cycle. **(B)** In *Bacillus subtilis*, DnaA indirectly inhibits the phosphorylation of Spo0A, a global regulator of sporulation and entrance to stationary phase.

DnaA is a multifunctional protein that can serve as a master regulator in bacteria. DnaA is composed of four structural domains with some species-specific variations: (I) protein–protein interaction domain, (II) linker domain, (III) AAA+ ATPase domain, and (IV) DNA binding (helix–turn–helix) domain (Fujikawa et al., 2003; Duderstadt and Berger, 2013) (Figure 1A). The two functions of DnaA that are best understood are as an initiator of chromosome replication and as a transcription factor. DnaA and/or the onset of chromosome replication have been linked to cell size regulation and chromosome segregation; however, details about these links remain unclear (Lobner-Olesen et al., 1989; Hill et al., 2012; Mera et al., 2014). As a replication initiator, DnaA opens the origin of replication (*ori*) by binding at specific DNA sequences referred to as DnaA boxes (Fuller and Kornberg, 1983; Bramhill and Kornberg, 1988). This function has been extensively studied, and mechanistic questions continue to be articulated (recent reviews; Hansen and Atlung, 2018; Frandi and Collier, 2019; Leonard et al., 2019; Ozaki, 2019; Kohiyama, 2020). In this mini review, we focus on the transcriptional activity of DnaA and its role in modulating various cellular events (Figure 1B). DnaA has been shown to have a global transcriptional impact because it regulates the expression levels of other global regulators involved in cell cycle progression and developmental processes (Figure 2). The specific genes found in DnaA’s transcriptional regulon vary significantly depending on the bacterial species. To begin, we will discuss the ability of DnaA to autoregulate its levels as a way to provide context for the importance of its transcriptional activity.

## Autoregulation of *DnaA* Expression

The ability of DnaA to autoregulate its transcription was discovered at a time when the direct role of DnaA in chromosome replication was not yet clear. Early characterizations of temperature-sensitive *dnaA* mutants of *Escherichia coli* revealed that the copy number of *ori* decreased, while DnaA levels increased simultaneously at nonpermissive temperatures, thus accurately predicting that DnaA had a positive involvement in the onset of chromosome replication and a negative involvement in regulating its own synthesis (Hanna and Carl, 1975; Hansen and Rasmussen, 1977). DnaA’s ability to repress its own transcription was later confirmed using *in vitro* and *in vivo* analyses. *In vitro*, DnaA was shown to directly bind *dnaA*’s promoter region using gel shift assays and DNase footprinting methods (Braun et al., 1985; Wang and Kaguni, 1987). *In vivo*, two strategies were used to show that DnaA represses its own transcription. First, increased cellular levels of DnaA (expressed from inducible promoters) were shown to decrease the activity of the *dnaA* promoter, and second, decreased DnaA levels able to bind the *dnaA* promoter (by adding *dnaA* binding sites on a plasmid that titrated DnaA levels away) were shown to increase the activity of the *dnaA* promoter (Atlung et al., 1985; Braun et al., 1985; Kucherer et al., 1986; Hansen et al., 1987).

In *E. coli*, the expression of *dnaA* is regulated from two promoters: *dnaAp1* and *dnaAp2* ((Hansen E. B. et al., 1982; Hansen F. G. et al., 1982) (Figure 1A). DnaA can repress its own expression from both promoters with *dnaAp2* being a threefold stronger promoter than *dnaAp1* (Atlung et al., 1984, 1985; Braun et al., 1985; Kucherer et al., 1986; Chiaramello and Zyskind, 1990). A 9-mer DnaA box with the consensus sequence TTATCCACA was first identified between *dnaAp1* and *dnaAp2* explaining the mechanism of autoregulation (Hansen F. G. et al., 1982). Intriguingly, the elimination of this DnaA box on *dnaA*’s promoter did not fully eliminate the autoregulation of *dnaA* expression, leading to the hypothesis of indirect mechanisms (Polaczek and Wright, 1990; Smith et al., 1997). The mystery was solved by the identification of DnaA-ATP-specific DnaA boxes (AGATCT) that were involved in full repression of *dnaA* expression (Speck et al., 1999). In addition to the autoregulation by DnaA, the transcription of *E. coli dnaA* is regulated at multiple levels and by various other proteins, including DNA methylation, second messenger ppGpp, growth rate, SeqA, Fis, IciA (ArgP), and QseB (Braun and Wright, 1986; Kucherer et al., 1986; Campbell and Kleckner, 1990; Chiaramello and Zyskind, 1990; Polaczek and Wright, 1990; Zyskind and Smith, 1992; Lu et al., 1994; Froelich et al., 1996; Lee et al., 1996; Hansen and Atlung, 2018; Riber and Lobner-Olesen, 2020; Wu et al., 2021).

Besides *E. coli*, autoregulation by DnaA directly binding at its promoter region has been confirmed in *Bacillus subtilis* (Ogura et al., 2001), *Vibrio harveyi* (Berenstein et al., 2002), *Streptomyces lividans* (Zakrzewska-Czerwinska et al., 1994; Jakimowicz et al., 2000), and in slow-growing mycobacteria (Salazar et al., 2003). In *Pseudomonas putida*, 12 DnaA boxes were identified on its *dnaA* promoter region (Fujita et al., 1989). However, when the levels of DnaA from *P. putida* were increased (by inducible expression from plasmid), repression of *dnaA* expression was not observed in *P. putida*. Interestingly, the overexpression of *dnaA* (only the open reading frame) from *E. coli* under the same broad host range plasmid construct in *P. putida* does result in repression of *dnaA*’s transcription (Ingmer and Atlung, 1992). The function, if any, of the 12 DnaA boxes in *P. putida dnaA* promoter remains to be determined. Likewise, in *Caulobacter crescentus*, the role that DnaA plays in its transcriptional regulation remains unclear. Transcription of *dnaA* in *C. crescentus* has been proposed to be regulated by methylation status of the promoter region and by a *cis-*acting element upstream of the -35 promoter region (Collier et al., 2007; Cheng and Keiler, 2009; Felletti et al., 2019; Frandi and Collier, 2019).

## Nucleotide Switch Regulates the Transcriptional Activity of DnaA

Both activities of DnaA (transcription factor and replication initiator) can be modulated using an ATP-dependent molecular switch. In *E. coli*, DnaA can be found inside the cell tightly bound to ATP (K_*d*_ 30 nM) or bound to ADP (K_*d*_ 100 nM) (Sekimizu et al., 1987). The protein Hda (homologous to DnaA) and chromosomal loci *datA* promote hydrolysis of DnaA–ATP to DnaA–ADP in *E. coli* (Kato and Katayama, 2001; Kasho and Katayama, 2013). The cellular ratio of DnaA–ATP to DnaA–ADP changes as the cell progresses over the cell cycle. Although DnaA–ATP and DnaA–ADP bind *ori*, only DnaA–ATP can open *ori* and initiate replication in *E. coli* (Sekimizu et al., 1987; Mizushima et al., 1996). As a transcription factor, the nucleotide switch has been shown to turn DnaA into a stronger repressor or stronger activator depending on the bound nucleotide. The ability of DnaA’s transcriptional activity to be regulated with this switch was first identified in the autoregulation of *dnaA*’s transcription in *E. coli* (Speck et al., 1999). DnaA–ATP is able to fully repress the expression of *dnaA*, whereas DnaA–ADP can only repress up to 40%. By distinguishing the nucleotide bound to DnaA, four new DnaA boxes were identified in the promoter region of *dnaA* with specificity for DnaA–ATP and potential cooperative binding (Speck et al., 1999).

The second example of this switch involves the synthesis of DNA substrates in *E. coli*. Ribonucleotide reductase (RNR) encoded by *nrdAB* catalyzes the reduction of ribonucleotides to deoxyribonucleotides (Thelander and Reichard, 1979). In *E. coli*, DnaA regulates the expression of *nrdAB* by binding the promoter region that contains three DnaA boxes: two boxes do not have specificity for the nucleotide-bound DnaA, and one box is specific for DnaA–ATP (Tuggle and Fuchs, 1986; Augustin et al., 1994; Jacobson and Fuchs, 1998; Olliver et al., 2010). DnaA can activate or repress the expression of *nrdAB* based on the nucleotide bound to DnaA and based on the levels of DnaA. For instance, cells expressing DnaA variants that are deficient for ATP binding or hyperactive for ATPase activity display an increased expression of *nrdAB* (Gon et al., 2006; Babu et al., 2017). Thus, high levels of DnaA-ATP repress the transcription of *nrdAB* presumably by precluding RNA polymerase from binding the promoter region (Olliver et al., 2010). Conversely, DnaA–ADP or low levels of DnaA–ATP bound at the high-affinity DnaA boxes in *nrdAB* promoter region can activate *nrdAB* transcription by stabilizing the RNA polymerase–DNA complex (Augustin et al., 1994; Olliver et al., 2010). Although the expression of RNR coincides with the onset of chromosome replication (Sun and Fuchs, 1992), DnaA seems to only regulate the expression levels and not necessarily the timing of *nrdAB* expression (Sun et al., 1994; Olliver et al., 2010). Interestingly, the expression of *dnaA* and *nrdAB* in *E. coli* share two other regulators besides DnaA: Fis and IciA (Augustin et al., 1994; Froelich et al., 1996; Lee et al., 1996; Han et al., 1998). In *B. subtilis* and *C. crescentus*, DnaA has also been proposed to act as a transcriptional regulator of genes encoding RNR (Goranov et al., 2005; Hottes et al., 2005), although no detailed analyses has been performed to determine if DnaA’s nucleotide switch is also involved.

In *C. crescentus*, DnaA-ADP (not DnaA-ATP) has been proposed to activate the transcription of a set of three essential genes encoding proteins involved in cell cycle regulation: FtsZ (tubulin-like protein essential for cell division), MipZ (inhibitor of FtsZ polymerization), and GcrA (global transcription factor) (Fernandez-Fernandez et al., 2011). The expression of the hyperactive replication initiator variant DnaA^*R357A*^ resulted in *C. crescentus* cells over-initiating replication (Fernandez-Fernandez et al., 2011; Wargachuk and Marczynski, 2015). The corresponding variant in *E. coli* (DnaA^*R334A*^) was shown to bind ATP but was unable to hydrolyze ATP (Nishida et al., 2002). In *C. crescentus*, DnaA^*R357A*^ variant was shown to lose its ability to activate the transcription of *ftsZ*, *mipZ*, and *gcrA* (Hottes et al., 2005; Fernandez-Fernandez et al., 2011). In *B. subtilis*, most chromosomal regions that bind DnaA displayed higher specificity for DnaA-ATP over DnaA-ADP *in vitro* (Smith and Grossman, 2015), suggesting a potential wide usage of DnaA’s nucleotide switch to modulate the transcriptional activity of DnaA. However, the mechanism(s) that regulate differences in DNA binding specificity between DnaA–ATP and DnaA–ADP remain unclear.

## DnaA’s Global Transcriptional Regulation

Identifying the complete transcriptional regulon of DnaA is complex due to the essential function of DnaA as a replication initiator and the role that replication initiation plays in the progression of the cell cycle. Work on *C. crescentus* and *B. subtilis* utilized innovative approaches to separate DnaA’s transcriptional activity from its function as a replication initiator (Hottes et al., 2005; Washington et al., 2017). One major finding from these whole-cell transcriptional analyses is that the transcriptional activity of DnaA can have a global effect on the cell. Aside from DnaA regulating the expression of various important genes, DnaA has also been found to be part of genetic networks where DnaA (directly or indirectly) regulates the expression of other transcription factors that are themselves global regulators (Figure 2). A global analysis of DnaA’s transcriptional activity remains to be determined for *E. coli*.

To identify the transcriptional regulon of DnaA in *C. crescentus*, Hottes et al. (2005) took advantage of the ability to synchronize the *Caulobacter* cell cycle in a strain with *dnaA*’s expression regulated from an inducible promoter. DnaA-dependent and DnaA-independent changes were identified by comparing transcriptional profiles of cells grown expressing *dnaA* with cells whose *dnaA* expression was delayed. This study focused on DnaA-dependent changes of genes whose expression increased during the transition from G1 to S phase. Of the 40 genes identified to be DnaA-dependent, 13 genes included DnaA boxes on their promoter region, and three genes (*gcrA*, *ftsZ*, and *podJ*) were shown *in vitro* to have promoter regions with affinity for purified His_6_DnaA. Notably, one of the DnaA-dependent genes identified was the gene encoding for GcrA, the global regulator of *Caulobacter* cell cycle (Holtzendorff et al., 2004) (Figure 2). The role of DnaA in the progression of the cell cycle has been predicted to be widely conserved among alpha proteobacteria (Panis et al., 2015). In the two alpha proteobacteria model systems *C. crescentus* (Hottes et al., 2005; Collier et al., 2006) and the plant symbiont *Sinorhizobium meliloti* (De Nisco et al., 2014), DnaA has been shown to be a key component of a closed genetic network that drives the progression through G1–S–G2.

In *B. subtilis*, the transcriptional regulon of DnaA has been characterized using various *in vitro* and *in vivo* high-throughput analyses (Goranov et al., 2005; Ishikawa et al., 2007; Cho et al., 2008; Breier and Grossman, 2009; Hoover et al., 2010; Smith and Grossman, 2015). To isolate the transcriptional activity of DnaA from DNA replication, Washington et al. (2017) eliminated DnaA’s essential role in replication by using a strain that initiates chromosome replication from the plasmids *oriN* by the plasmids replication initiator RepN. This *B. subtilis* strain has its native *ori* and the operon flanking it (*dnaA–dnaN*) knocked out. The expressions of *dnaA* and *dnaN* were engineered at different loci regulated by two different inducible promoters. Using this system, 91% of 339 total number of genes that displayed DnaA-dependent transcriptional regulation were shown to be indirectly regulated by DnaA via Sda. DnaA regulates the transcription of *sda* by directly binding at *sda*’s promoter region (Burkholder et al., 2001; Ishikawa et al., 2007; Breier and Grossman, 2009). Sda is an inhibitor of the phosphorelay that ultimately activates Spo0A, a global regulator of sporulation and stationary phase gene expression (Figure 2B) (Burkholder et al., 2001; Rowland et al., 2004; Whitten et al., 2007). Consistent with previous analyses, Washington et al. identified eight sets of genes that are directly regulated by DnaA: *dnaA-dnaN*, *sda*, *yqeG-M*, *ywlC*, *ywcI-sacT*, *vpr*, *yyzF-yydABCD*, and *trmEF-rsmG-noc* (Burkholder et al., 2001; Ogura et al., 2001; Ishikawa et al., 2007; Washington et al., 2017).

## Transcriptional Activity of DnaA and Its Role in DNA Replication

### Replication Initiation

Aside from opening the double-stranded *ori* region, DnaA has been proposed to regulate the initiation of replication in three other ways that are dependent on its transcriptional activity. First, DnaA regulates the levels of the replication initiator by modulating its own expression in various bacterial species (as discussed in previous sections). Second, in *C. crescentus*, DnaA regulates the levels of the active form of the replication initiator (DnaA–ATP) by regulating the expression of *hdaA* (Collier and Shapiro, 2009). HdaA represses DnaA’s activity as a replication initiator by promoting the hydrolysis of DnaA–ATP to DnaA–ADP (Collier and Shapiro, 2009). Third, DnaA’s transcriptional activity can promote the replication of λ-derived plasmids via a potential direct interaction between DnaA and the β-subunit of RNA polymerase (RpoB) (Szalewska-Palasz et al., 1998b). In *E. coli*, DnaA’s binding at *ori-*λ is necessary for transcriptional activation of *ori-*λ and also for efficient expression of λ replication initiator proteins (Szalewska-Palasz et al., 1998a).

### DNA Replication

DnaA as a transcription factor regulates the levels of substrates for DNA synthesis and of components of the replisome (molecular machinery required for chromosome replication). In *E. coli*, DnaA has been shown to repress the expression of the *gua* operon involved in purine biosynthesis (Tesfa-Selase and Drabble, 1992). The role of DnaA in regulating expression levels of *nrdAB* (involved in the last step of deoxynucleotide biosynthesis) has been shown in *E. coli* and proposed in *C. crescentus* (Tuggle and Fuchs, 1986; Augustin et al., 1994; Jacobson and Fuchs, 1998; Hottes et al., 2005). In *E. coli* and *B. subtilis*, DnaA regulates the transcription of *dnaN* (DNA polymerase III, β-subunit) commonly found in the same operon downstream of *dnaA* (Hansen F. G. et al., 1982; Ogura et al., 2001; Berenstein et al., 2002; Washington et al., 2017). In *C. crescentus*, predicted DnaA boxes are found upstream of *dnaQ* (DNA polymerase III, β-subunit) and *dnaB* (DNA helicase), both of which were identified as part of the DnaA transcriptional regulon by delaying *dnaA* transcription (Hottes et al., 2005).

### DNA Repair

DNA repair plays an important role in DNA replication due to the relatively frequent replication-fork arrests that occur in bacteria even when growing under normal conditions (Cox et al., 2000). DnaA has been proposed to serve as a regulator for maintaining the integrity of the genome in response to DNA damage (Wurihan et al., 2018). In stationary phase, DnaA activates the transcription of *polA* (Quinones et al., 1997), the DNA polymerase in *E. coli* involved in DNA replication and DNA repair (Sharma and Smith, 1987; Savic et al., 1990; Quinones et al., 1997). DnaA along with LexA (global regulator of the SOS response) were shown to co-regulate the transcription of two key genes in the SOS regulon (Wurihan et al., 2018): *uvrB* (component of Nucleotide Excision Repair NER system) and *recN* [involved in repair of double-stranded breaks (Uranga et al., 2017)]. The expression of *uvrB* had previously been predicted to be regulated by DnaA based on DnaA boxes found upstream of *uvrB* (van den Berg et al., 1985; Arikan et al., 1986). In *B. subtilis*, the transcriptional activity of DnaA has been linked to the RecA-independent response to DNA stress (Goranov et al., 2005; Washington et al., 2017).

## DnaA Beyond Chromosome Replication

### Cytokinesis

Maintaining the integrity of the chromosome after each cell division requires exquisite coordination between chromosome replication and cytokinesis. In *E. coli*, the expression levels of the gene encoding FtsZ oscillate over the cell cycle reaching the highest levels at the same time when chromosome replication initiates (Garrido et al., 1993). The cell cycle-dependent expression of *ftsZ* and the identification of three DnaA boxes found upstream of *ftsZ* (within *ftsQA*) initially suggested DnaA’s involvement in *ftsZ* expression; however, the three DnaA boxes were later shown not to play a role in *ftsZ* transcription (Masters et al., 1989; Garrido et al., 1993; Smith et al., 1996). A more recent potential connection between DnaA’s transcriptional activity and cytokinesis involves MioC in *E. coli* in that DnaA regulates the transcription of *mioC* that is located next to *ori* (Lother et al., 1985; Rokeach and Zyskind, 1986; Nozaki et al., 1988). The protein MioC has been proposed to promote cell division independent of *ori*’s replication and segregation (Lies et al., 2015). However, *mioC* mutants display only a moderate cell division defect. In *C. crescentus*, DnaA was shown to bind *ftsZ*’s promoter region *in vitro* and its *in vivo* expression to be dependent on DnaA’s nucleotide switch (Hottes et al., 2005; Fernandez-Fernandez et al., 2011). In *B. subtilis*, DnaA was shown to bind the promoter region of *ftsL* (encodes membrane-associated Z-ring protein) *in vivo* using chromatin immunoprecipitation assays (Goranov et al., 2005). So far, the transcriptional activity of DnaA has not been shown to have a major impact with the timing of cytokinesis in any bacterial species. Our current understanding of the mechanism(s) that coordinate the timing of replication initiation with cytokinesis remains limited.

### Motility, Quorum Sensing, and Heat Shock Response

The transcriptional activity of DnaA has been linked to other developmental processes. The first observation connecting DnaA to motility was in a temperature-sensitive *dnaA* mutant of *E. coli*. When grown at its permissive temperature, this mutant strain expressed significantly lower levels of flagellin that resulted in loss of motility (Mizushima et al., 1994). DnaA’s regulation was later shown to not directly affect the expression of flagellin but rather indirectly via the expression of the gene (*flhD*) encoding the flagellar transcriptional regulator FlhD (Mizushima et al., 1997). Notably, the quorum sensing regulators QseB and QseC in *E. coli* have been shown to regulate the expression of *dnaA* and *flhD* (Sperandio et al., 2002; Wu et al., 2021). In the nitrogen-fixing symbiont *S. meliloti*, the transcriptional activity of DnaA has been proposed to coordinate growth phase with quorum sensing. DnaA was shown to activate the transcription of *nurR* (encodes a LuxR-like solo regulator) by binding the promoter region during exponential growth and high nutrient availability (McIntosh et al., 2019). NurR activates the transcription of *sinR*, which encodes the major regulator of *N*-acyl-homoserine lactones (AHL) production (Calatrava-Morales et al., 2018). The transcriptional activity of DnaA has also been connected to *E. coli* heat shock response. DnaA was shown to bind the promoter region and repress the transcription of the gene encoding the sigma factor RpoH (Wang and Kaguni, 1989).

## Future Directions for Replication Initiators

This review has focused on DnaA as the key chromosome replication initiator in bacteria. Replication initiators are different in the other domains of life such as archaea and eukaryotes. Interestingly, the replication initiators in archaea and in eukaryotes have also been shown to serve as regulators of gene expression. In eukaryotes, chromosome replication is initiated by the origin recognition complex (ORC) composed of six subunits (Bell and Dutta, 2002). In human cells, replication initiator proteins have been shown to regulate the transcription of genes involved in cell division (Hossain and Stillman, 2016). Replication initiation in archaea resembles the molecular machinery of ORC in eukaryotes (Makarova and Koonin, 2013). In the archaeon *Sulfolobus islandicus*, replication initiator proteins were shown to bind the promoter regions and regulate the expression of genes involved in DNA damage response (Sun et al., 2018). The ability of replication initiators to regulate gene expression in eukaryotes and archaea are a relatively recent discovery that continues to accumulate supporting data (Popova et al., 2018; Hu et al., 2020).

In this mini review, several aspects of the transcriptional activity of DnaA were discussed including autoregulation, its mechanism of regulation, and its role in chromosome replication and other key cellular events. Many exciting questions about the transcriptional activity of DnaA remain to be answered. For instance, DnaA has been shown to act as an activator and a repressor of transcription (Messer and Weigel, 1997). Is there a common mechanism that differentiates and regulates these two opposing functions of DnaA? Furthermore, bipartite *oris* flanking the *dnaA* gene (as in *B. subtilis* and *Helicobacter pylori*; Krause et al., 1997; Donczew et al., 2012) suggest that DnaA can act as a replication initiator and transcription factor when bound at the same chromosomal locus upstream of *dnaA*. How are these two different functions of DnaA coordinated and differentiated over the cell cycle? Most of the work done on DnaA’s nucleotide switch has been performed in *E. coli*. How widespread is DnaA’s nucleotide switch used to regulate DnaA’s transcriptional activity in other bacterial species? To conclude, the transcriptional activity of DnaA has only been characterized in a few bacterial species. Within these few, the specifics of DnaA’s transcriptional regulon vary significantly. Thus, the characterization of replication initiators’ roles in coordinating various cellular events will continue to provide exciting results for years to come.

## Author Contributions

All authors contributed to the article and approved the submitted version.

## Conflict of Interest

The authors declare that the research was conducted in the absence of any commercial or financial relationships that could be construed as a potential conflict of interest.
